# Changes in Structure and Histochemistry of Glandular Trichomes of *Thymus quinquecostatus* Celak

**DOI:** 10.1100/2012/187261

**Published:** 2012-04-01

**Authors:** Ping Jia, Ting Gao, Hua Xin

**Affiliations:** College of Life Sciences, Qingdao Agricultural University, Qingdao 266109, China

## Abstract

The types, morphology, distribution, structure, and development process of the glandular trichomes on the leaves of *Thymus quinquecostatus* Celak had been investigated in this study. Two different types of glandular trichomes were determined in detail, namely, capitate trichomes and peltate ones. Besides, there were distinct differences on morphology, distribution, structure, and development process between the two kinds of trichomes. As the peltate trichome stepping into senium stage, it caved in the epidermis integrally, which was different from the capitate one. The secretion of the capitate trichome contained essential oil, polyphenols, and flavonoids, while, in addition to these three components, the secretion of the peltate one also contained acid polysaccharides. A distinctive difference was also seen in the secretory pathway of the secretion between the two types of trichomes. The secretion of capitate one was extruded through the cuticle of the head cell, but the secretion of the peltate one kept accumulating in the subcuticular space of the head cells until it was released by cuticle rupture.

## 1. Introduction

*T. quinquecostatus* is a scrubby subshrub of *Thymus *Lamioideae Lamiaceae, and the whole plant is highly aromatic. As a traditional Chinese medicine, it has anti-inflammatory and analgesic effects. The essential oil of *Thymus* plants already widely studied is a kind of natural perfume, and it has a high economic value because it is used to produce cosmetic fragrance and edible essence. Fifty-one constituents were identified in the essential oils of *Thymus transcaspicus* [1], thirty-four and fifty-three constituents were identified in the essential oils of* T. quinquecostatus *and *T. mongolicus,* respectively [2, 3]. The in vitro inhibitory activities of essential oils from *T. magnus* and *T. quinquecostatus* as well as their main constituents were evaluated against susceptible and resistant species of *Streptococcus pneumoniae*,* Staphylococcus. aureus*, *Salmonella enteritidis,* and *Salmonella typhimurium* [4]; moreover, the effect on resisting tumour of the ethanol extracts from *T. quinquecostatus* was obvious [5].

 Glandular trichomes are generally considered as the site of biosynthesis and accumulation of essential oil [6, 7]. As an exocrine structure, the glandular trichomes are widely distributed over the aerial organs of the plants, which is one of the characteristic features of Lamiaceae. Abundant studies on glandular trichomes of Lamiaceae were carried out [8–10], while, so far, few studies on trichomes of *T. quinquecostatus* were reported. Therefore, we studied the types, distribution, structure, development process, and secretion of the glandular trichomes present on leaves of *T. quinquecostatus*.

## 2. Materials and Methods

### 2.1. Plant Materials

 From April to July of 2010, bud, young leaf and mature leaf samples of *T. quinquecostatus* were collected from plants growing in the experimental field at Qingdao Agricultural University.

### 2.2. Methods

#### 2.2.1. Semithin Sections

1-2 mm^2^ small pieces of the materials were fixed in paraformaldehyde and 25% glutaraldehyde in 0.2 M phosphate buffer, washed by 0.1 M phosphate buffer for 4 times and 20 minutes for each time, and then dehydrated in ethanol in ascending grades up to absolute, then activated in the activator for 3–5 days, finally embedded in the Technovit 7100. 2 *μ*m pieces were sectioned with an AO-820 Rotary Microtome. The sections were stained by hematoxylin after they were dried completely and observed with a Leica DM2500 microscope.

#### 2.2.2. SEM Observations

Bud and leaf samples were fixed in paraformaldehyde and 25% glutaraldehyde, and after critical point drying, sprayed a metallic luster in the vacuum evaporator, finally observed and photographed with a KYKY-2800B SEM.

#### 2.2.3. Histochemical Methods

Leaf samples were embedded in agar (4%) and sectioned (20 *μ*m) with a MICROM HM525 freezing microtome. The fresh sections were stained by the following histochemical tests: (1) Sudan III and Sudan black B, for essential oil; (2) nile blue, for neutral and acidic lipids; (3) ferric trichloride (FeCl_3_) and toluidine blue, for polyphenols; (4) Alcian blue, for acid polysaccharides; (5) aluminum trichloride (AlCl_3_), for flavonoids. The stained sections were observed and photographed by a Leica DM2500 microscope.

## 3. Results

### 3.1. Types and Distribution of Glandular Trichomes

**Figure 1:**
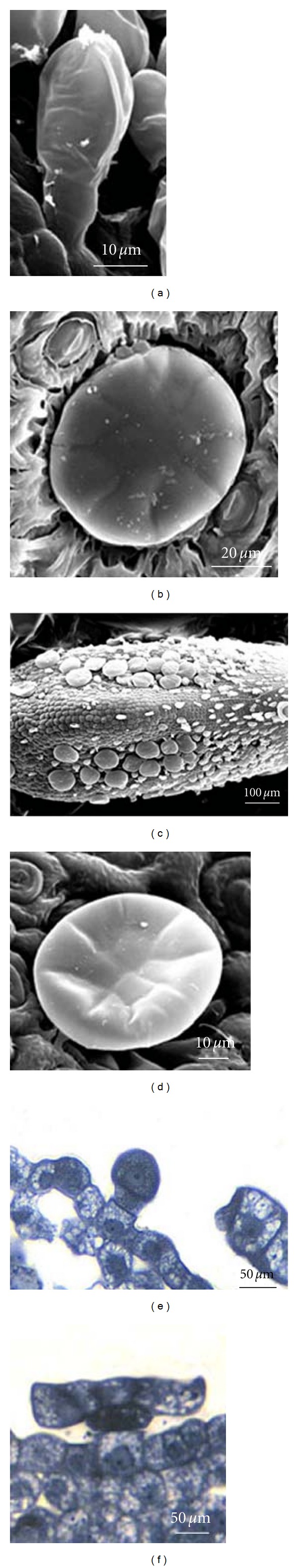
(a–d) SEM micrographs showing the morphology of glandular trichomes on leaves of *T. quinquecostatus.* (a) A capitate trichome. (b) A peltate trichome. (c) The abaxial leaf surface, showing the difference of the distribution between the two kinds of glandular trichomes. (d) The head of the peltate trichome. (e-f) The transverse sections of *T. quinquecostatus* leaves (e) showing a mature capitate trichome. (f) A peltate trichome.

The glandular trichomes on the surface of leaves are of two types, peltate and capitate. The two types are very different in morphology and distribution. Capitate trichomes have a short stalk and a round head (Figure 1(a)); the peltate trichomes have a large disc-like head and a very short stalk (Figure 1(b)). Two kinds of glandular trichomes distribute both on adaxial and abaxial surfaces of the leaf. Furthermore, the peltate trichomes are only located on the intercostals area, while the capitate trichomes exist everywhere on the leaf, especially on the midrib (Figure 1(c)).

### 3.2. Composition of Glandular Trichomes

Besides the differences of morphology, distinction between the two types of glandular trichomes is also very obvious, which is often related to the mechanism of the essential oil releasing.

The capitate trichomes consisted of a basal cell, a stalk cell, and a unicellular head (Figure 1(e)). The elongated basal cell is a little bigger than the contiguous epidermal cells of the leaf. The stalk cell with thickened walls is usually short in length, and it is adjacent to the head. The head only has one secretory cell.

The formation of peltate trichome is similar to the capitate one, which contains a basal cell, a stalk cell, and a head (Figure 1(f)). Nevertheless, the head of peltate trichome is greatly distinguished from that of capitate trichome. It has 12 secretory cells forming two cycles, which includes 4 central and 8 peripheral cells (Figure 1(d)). Owing to the arrangement of the secretory cells, the head presented a peltate shape.

### 3.3. Development Process

The capitate and peltate trichomes both originate from the procuticle cell. But, even so, the differences of the development process between the two types of glandular trichomes are distinct. Moreover, the essential oil would be secreted, accumulated, and released following the development of the glandular trichomes.

#### 3.3.1. The Development of Capitate Trichome

**Figure 2:**
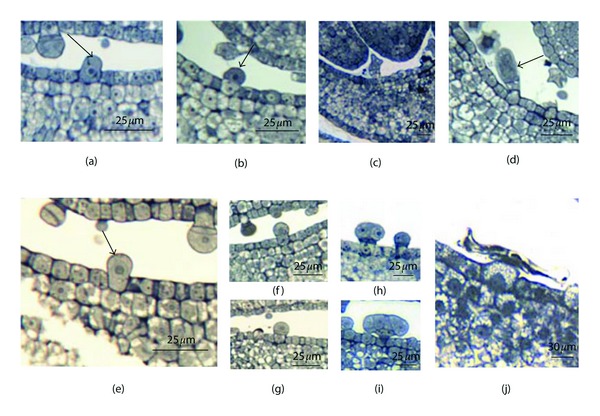
Transverse sections of *T. quinquecostatus* leaves in different stages. (a–d) show the whole development process of capitate trichome. (a) The initial cell of capitate trichome. (b) Two cells after periclinal division of initial cell. (c) Three cells after two periclinal divisions. (d) A mature capitate trichome. (e–j) show the development process of peltate trichome. (e) The initial cell of peltate trichome. (f) Two cells after periclinal division of initial cell. (g-i) The formation of head cells. (j) A mature peltate trichome.

The precursor of glandular trichome is an enlarged procuticle cell with dense cytoplasm and increscent nucleus (Figure 2(a)). In the early stage of development, the enlarged procuticle cell protrudes above the leaf epidermis and the nucleus also removes towards the top of the procuticle cell, thus the cell forms a polarization leading to the first asymmetric periclinal division which would produce a basal cell and an upper one (Figure 2(b)). The basal cell would no longer divide, while the upper one would undergo a periclinal division further, after the second division, a capitate trichome containing a basal cell, a stalk cell, and a secretory cell has completed (Figure 2(c)). The stalk cell is cylindrical, but it flats slightly when the capitate trichome matures (Figure 2(d)).

#### 3.3.2. The Development of Peltate Trichome

**Figure 3:**
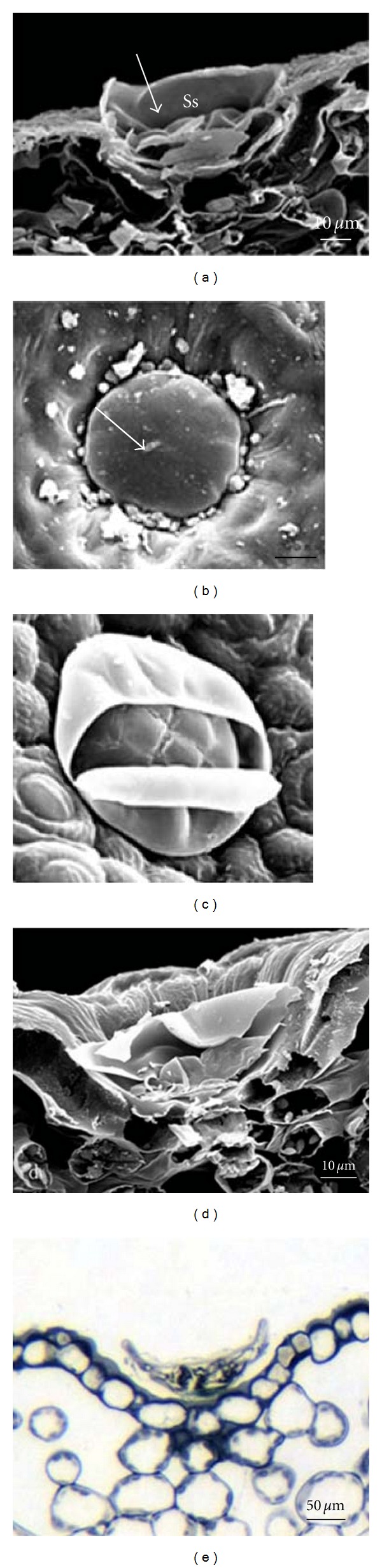
(a–d) SEM micrographs showing the morphology of peltate trichomes on leaves of *T. quinquecostatus. *(a) The vertical section of peltate trichome, showing the subcuticular space. (b-c) show the cuticle rupture through which the essential oil released. (d) The senescent peltate trichome caving in to the epidermis entirely. (e) The semithin section stained by hematoxylin, showing the senescent peltate trichome.

The beginning development process of the peltate trichome is as same as that of capitate one until the first periclinal division finished. The two cells formed by the first division are almost the same in size (Figures 2(e) and 2(f)), the bottom one develops into the basal cell and the upper one continues to divide. The upper cell divides into two different-sized daughter cells by a second asymmetric periclinal division (Figure 2(g)). In the two daughter cells, the applanate one evolves into the stalk cell, which borders upon the basal cell, the other daughter cell finally divides into 12 secretory cells by divisions (Figure 2(h)–2(j)). Along with the peltate trichome maturing, the cuticle separates from the cell walls of secretory cells, thus a large subcuticular space in which the essential oil stored forms (Figure 3(a)). The essential oil accumulates in the subcuticular space day after day, and finally it is released by cuticle rupture (Figures 3(b) and 3(c)). As the essential oil releasing, the peltate trichome sinks gradually. Finally, it caves in the epidermis integrally (Figures 3(d) and 3(e)).

In the senium stage of the peltate trichomes, two phenomenons are remarkable. Firstly, the stalk cell is of cutinization at the time of essential oil accumulating; moreover, the cell is more flat. Secondly, the epidermal cells around peltate trichomes cave obviously with the glandular trichome sinking below the epidermis.

### 3.4. Histochemical Analysis

#### 3.4.1. The Essential Oil

The sections are stained positive with Sudan III and Sudan black B. The results demonstrated that the secretions of the two kinds of glandular trichomes both contain essential oil, but they were obviously distinct in secretory pathway.

**Figure 4:**
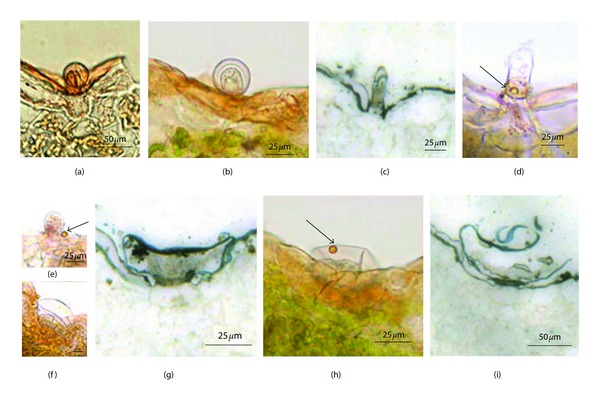
(a, b, d–f, h) The frozen sections of leaves of *T. quinquecostatus* stained with Sudan III. (c, g, i) The semithin sections of leaves of *T. quinquecostatus* stained with Sudan black. (a-b) The capitate trichome stained orange-yellow. (c) The capitate trichome was stained gray black. (d) The oil drop in the stalk cell of capitate trichome. (e) The oil drop was secreted out of the capitate trichome. (f) The peltate trichome was stained orange-yellow. (g) The peltate trichome was stained gray black. (h) An oil drop in the subcuticular space of peltate trichome. (i) A peltate trichome with the ruptured cuticle.

The capitate trichome was stained orange-yellow with Sudan III (Figures 4(a) and 4(b)) and black with Sudan black B (Figure 4(c)). The small droplets that stained orange-yellow with Sudan III presented in the stalk cell (Figure 4(d)). Finally, the lipid drops seemed to be extruded through the cuticle (Figure 4(e)). The peltate trichome was also stained orange-yellow with Sudan III (Figure 4(f)) and black with Sudan black B (Figure 4(g)), which indicated that the essential oil existed in the peltate trichome and accumulated in the subcuticular space after it was secreted (Figure 4(h)), and, at last, it was released by cuticle rupture (Figure 4(i)).

**Figure 5:**
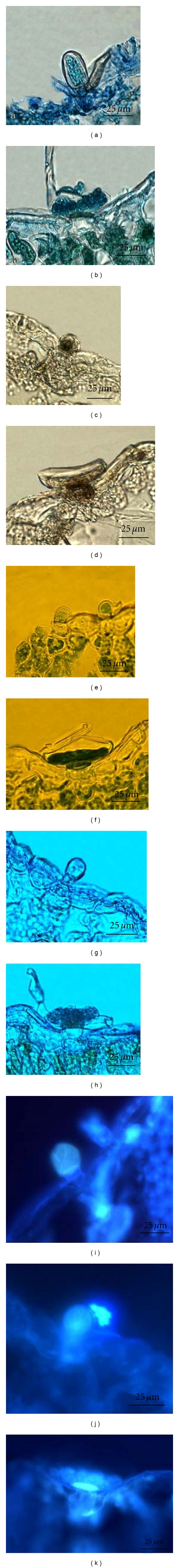
The sections of leaves of *T. quinquecostatus.* (a) A capitate trichome stained with nile blue. (b) A peltate trichome stained with nile blue. (c, d) show a capitate trichome (c) and peltate one (d) stained with FeCl_3_. (e, f) show a capitate trichome (e) and peltate one (f) stained with Toluidine blue. (g, h) show a capitate trichome (g) and peltate one (h) stained with Alcian blue. (i, j) Capitate trichomes stained with AlCl_3_ under fluorescence microscope. (k) A peltate trichome stained with AlCl_3_ under fluorescence microscope.

Nile blue was used to distinguish neutral lipids from acidic lipids, the former being colored red, the latter dark blue. After dyeing treatment of sections, the capitate and peltate trichomes both presented to be dark blue, without being red obviously (Figures 5(a) and 5(b)). The existence of acidic lipids was clearly proved, but the neutral lipids did not exist in the secretion.

#### 3.4.2. The Polyphenols

FeCl_3_ and toluidine blue are usually used to detect polyphenols, which are colored brown by FeCl_3_ and green by Toluidine blue. In the capitate trichome, the head cell was stained brown by FeCl_3_ while the basal cell and stalk cell were not colored (Figure 5(c)). In contrast, all cells of peltate trichome were strongly colored by FeCl_3_, and especially the stalk cell (Figure 5(d)). After the Toluidine blue staining, just the head cell of the capitate trichome was dyed green but the stalk cell and basal cell were not (Figure 5(e)); both stalk cell and head cells of peltate trichome were colored green, but basal cell was not (Figure 5(f)). Thus, the polyphenols did not only exist in the capitate trichome but also existed in the peltate one.

#### 3.4.3. The Acid Polysaccharides

Alcian blue is used to detect acid polysaccharides, which could be stained blue with it. For the capitate trichome, the cell wall of the head cell was stained blue obviously (Figure 5(g)). It is possible that it was related to the pectin of cell wall. The stalk cell and head cells of peltate trichome were colored blue, while the basal cell remained unstained (Figure 5(h)), denoting the acid polysaccharides in the secretion.

#### 3.4.4. The Flavonoids

AlCl_3_ is used to detect flavonoids, which present blue under the fluorescence microscope. Both the capitate and peltate trichome were stained blue with AlCl_3_ (Figures 5(i)–5(k)), denoting the flavonoids in the secretion.

## 4. Discussion

According to the current literature, there are two types of glandular trichomes, capitate and peltate, in plants of Lamiaceae. The types and distribution of glandular trichomes make a great deal of difference among different species. Therefore, the glandular trichomes are regarded as an effective character in the subfamily classification of Lamiaceae, some species just have one type while other species may have two types. Giuliani and Maleci Bini studied the glandular trichomes of several species belonging to subfamily Lamioideae, a kind of large capitate trichome was discovered as well besides capitate and peltate ones [8]. *T. quinquecostatus* also belongs to subfamily Lamioideae, capitate and peltate trichomes were discovered on the leaf surface, which was similar to the types of trichomes in *Stachys alopecuros* subsp. *alopecuros*, *Stachys officinalis* subsp. *officinalis*, *Scutellaria galericulata,* and *Mentha haplocalyx*.

 Two types of glandular trichomes both contain a basal cell, a stalk cell, and a head, but they have significant morphological differences. The capitate one just has one secretory cell, so it presents an oval shape; the peltate one presents a disc-like shape as a result of the arrangement of 12 secretory cells. Generally speaking, they distribute on both sides of the leaf, but, in detail, capitate trichomes usually spread on the leaf surface especially on the veins, while the peltate ones just exist on intercostals area. So far, most researches studied on the distribution mainly focusing on differences between abaxial and adaxial surfaces of leaf, while the studies about the detailed distribution on intercostals area and veins were very few.

The origination and early development of the two types of glandular trichomes is similar. The initial cells of two types of glandular trichomes originate from the protocuticle cells and create a basal cell, a stalk cell, and an apical cell through two successive periclinal divisions. After that, the development of glandular trichomes have two circumstances: (1) the apical cell no longer divides, then the three cells develop into capitate trichome directly; (2) the apical cell still has the capacity of cell division, afterwards a big head with 12 secretory cells forms, and finally a peltate trichome is completed. When it matures, the peltate trichome forms a big subcuticular space, while the capitate one did not. After the glandular trichomes come into secretion stage, the cutinization of the stalk cell occurs and its cell wall becomes thickened. Some researchers discovered that the cutinized stalk cell formed a barrier against the free movement of substances between the glandular head and the leaf through apoplasts [10]. In the previous studies on plants of Lamiaceae, most peltate trichomes fell off from the mature leaves; however, in our studies, with the maturing of leaves of *T. quinquecostatus*, rather than falling off, the peltate trichomes sunk gradually and finally sunk under the epidermis completely, which had not been reported in other literatures. And we discovered that the essential oil existed still on old leaves of *T. quinquecostatus*. Whether it deposited on the surface of peltate trichomes after being secreted or the secretory cells kept secreting when the cuticle broke needed a further study.

Different secretory pathways of glandular trichomes of Lamiaceae were discovered, the secreting material seemed to be extruded through the cuticle or released through cuticle rupture or through the apical pore of secretory cells [8, 11, 12]. In our studies, two types of secretory pathways were discovered. In the capitate trichomes, secreting material was extruded through the cuticle; while in the peltate ones, the secreting material kept on accumulating in the subcuticular space and finally released by cuticle rupture.

The secretion of Lamiaceae mainly contains two kinds of materials, those are essential oil and polysaccharide. The peltate trichomes mainly secrete essential oil, while the secretion of capitate trichomes also contained polysaccharide besides essential oil [10]. In our research, capitate and peltate trichomes of *T. quinquecostatus* both contain essential oil, which is similar to the result of most other researchers. But, after staining with Alcian blue, the stalk cell and head cells were colored blue, denoting the existence of polysaccharide in the secretory of peltate trichomes; while in the capitate trichomes, just the surface of head cell was blue, which was related to the pectin of the cell wall. Furthermore, both capitate and peltate trichomes contain polyphenols and flavonoids according to the histochemical methods. How to produce these kinds of secretions in glandular trichomes? Secretory pathways in their cells need to be further studied.
